# The Ocular Surface Chemical Burns

**DOI:** 10.1155/2014/196827

**Published:** 2014-07-01

**Authors:** Medi Eslani, Alireza Baradaran-Rafii, Asadolah Movahedan, Ali R. Djalilian

**Affiliations:** ^1^Department of Ophthalmology and Visual Sciences, University of Illinois at Chicago, Chicago, IL 60612, USA; ^2^Labbafinejad Medical Center, Shahid Beheshti University of Medical Sciences, Tehran, Iran

## Abstract

Ocular chemical burns are common and serious ocular emergencies that require immediate and intensive evaluation and care. The victims of such incidents are usually young, and therefore loss of vision and disfigurement could dramatically affect their lives. The clinical course can be divided into immediate, acute, early, and late reparative phases. The degree of limbal, corneal, and conjunctival involvement at the time of injury is critically associated with prognosis. The treatment starts with simple but vision saving steps and is continued with complicated surgical procedures later in the course of the disease. The goal of treatment is to restore the normal ocular surface anatomy and function. Limbal stem cell transplantation, amniotic membrane transplantation, and ultimately keratoprosthesis may be indicated depending on the patients' needs.

## 1. Introduction

A chemical ocular burn usually occurs when a corrosive substance is accidentally introduced to the eye and/or periocular tissues. Chemical burn is considered a true ocular emergency and requires immediate and intensive evaluation and care. This type of injury is most common among men 20 to 40 years of age that typically work in industrial chemical laboratories or factories [1]. Given their younger age, the long-term disabilities that follow ocular burns could dramatically affect the patients' lives. The goal of treatment is to minimize further damage to ocular surface and ultimately restore a normal ocular surface anatomy and visual function.

## 2. Presentation

The typical presentation after a chemical injury is a sudden onset of severe pain, epiphora, and blepharospasm [2]. Basic substances are lipophilic and penetrate the eye more rapidly compared to acidic chemicals. They may also find their way to the anterior chamber damaging the trabecular meshwork, ciliary body, and the lens. Due to the rapidity of this process, patients may experience irreversible intraocular damage in as little as 5–15 minutes [2]. Acid injuries tend to be less severe. Acids cause protein coagulation in the epithelium, which limits further penetration into the deeper layers of the eye (hydrofluoric acid is an exception among acid since it can rapidly pass through cell membranes) [3–5]. Shrinkage and contraction of the cornea and sclera may lead to acute rise of intraocular pressure. Long-term rises of intraocular pressure can occur from fibrotic damage to the trabecular meshwork as well as the inflammatory debris trapping within the meshwork [1–3]. Conjunctival inflammation and loss of goblet cells can leave the ocular surface prone to dryness, scarring, and contracture of the fornices [2, 3, 6, 7].

## 3. Clinical Examination

**Figure 1:**
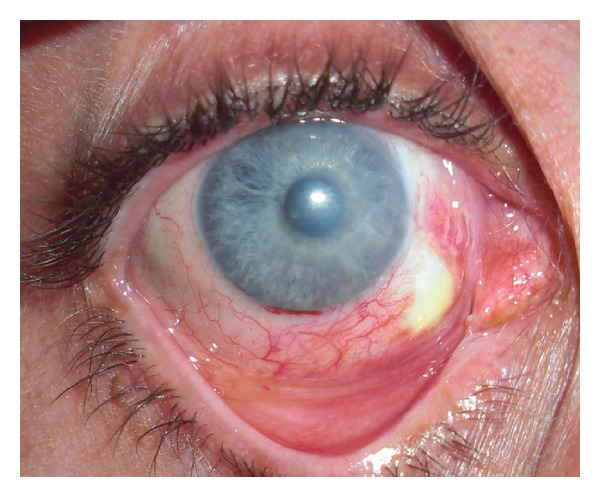
Limbal ischemia in the inferonasal quadrant 8 days after alkali burn. Patient subsequently underwent tenonplasty and conjunctival advancement to cover the defect.

**Figure 2:**
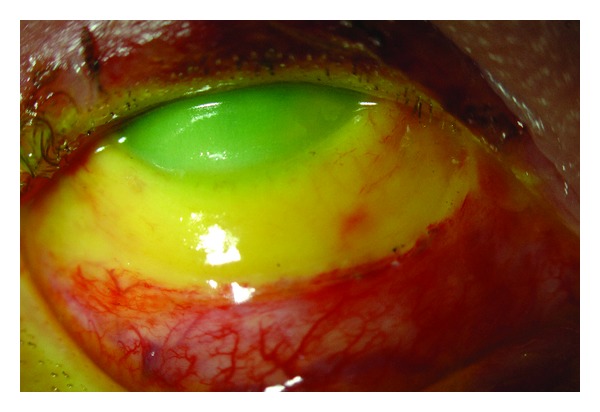
Patient with grade IV ocular surface burn. Note severe ischemia extending 4 mm from the cornea and corneal haze. Patient required multiple reconstructive procedures including combined conjunctival-limbal autograft and keratolimbal allograft.

The initial examination (after thorough irrigation as described below) includes a complete eye examination [1, 2, 7]. It is essential to make sure that no foreign bodies are embedded in any part of the ocular structures. A spectrum of clinical manifestations after a chemical injury could be described, which may vary substantially over time. Acute periocular signs of injury include periorbital edema and erythema, deepithelialized skin, and loss of eyelashes and eyebrows. Early signs include corneal and conjunctival epithelial defects, chemosis, conjunctival inflammation, limbal ischemia (Figure 1), corneal cloudiness, sterile ulceration, edema, and occasionally perforation [1, 7]. High intraocular pressure may result from damage and/or inflammation of the trabecular meshwork. One of the most important prognostic factors for the visual outcome is the extent of ocular surface damage, initially reflected by the amount of limbal ischemia (Figure 2) [1–3, 6–8]. Extensive damage to the limbus leads to limbal stem cell deficiency (LSCD) which may ultimately result in failure of normal corneal epithelial healing, neovascularization, and conjunctivalization. Lagophthalmos can also interfere with reepithelialization; it may be secondary to mechanical changes in the lids, secondary to edema or scarring. Extensive conjunctival burns can lead to long-term consequences including symblepharon, cicatricial entropion and ectropion, and trichiasis that may further complicate the presentations [2, 9–14].

## 4. Classification

Identifying the stage of a chemical eye burn is particularly helpful in prediction of the outcome. Most importantly, the relative proportion of surviving limbal tissue has been shown to be a major prognostic factor [1–3, 6–8]. Several classifications have been proposed [15–17]: the Roper-Hall classification system was initially developed in the mid 1960s, first by Ballen [18], and then modified by Roper-Hall [17]. The basis of this classification was largely on the degree of corneal haze and the amount of perilimbal ischemia (Table 1). Pfister subsequently presented a classification system grading the injury from mild, mild-moderate, moderate to severe, severe, and very severe based upon photographs demonstrating corneal haze and perilimbal ischemia [15]. Dua proposed a classification scheme based on clock hour limbal involvement (versus ischemia) as well as percentage of bulbar conjunctival involvement [16]. Overall, the key element is to note the amount of limbal, corneal, and conjunctival involvement at the time of injury [1].

## 5. Pathogenesis

The typical pathophysiological course of events starts with a sudden change of tissue pH followed by pH-dependent chemical alterations [19, 20]. Until recently, the chemical characteristics including pH of the hazardous agent have been considered as the key element in determining the amount and type of tissue damage [19]. However, it has been shown that other factors such as temperature, amount, impact force, concentration, dissociation coefficient (e.g., osmolarity), redox-potential, and specific reactivity with the ocular tissues (pK values) can greatly influence the pathophysiologic cascade of chemical tissue damage [3].

The temperature determines nonspecific coagulation or cooling of the tissues. A hot solution generally causes more damage than a similar cool solution, since the chemical reactivity usually increases with a rise in temperature [3]. Solid substances are not removed by blinking, and corrosive powders such as lime or concrete may remain in greater concentration in conjunctival sac and thus have higher chances to destroy the tissues. Lime particles in particular may cause severe ongoing damage if they remain unnoticed in the deep fornices [3, 21]. The impact force of a corrosive agent is also noteworthy [3]; it influences the amount of corrosive substance placed on the cornea and in the conjunctival sac as well as the tissue reactivity after the accident. Low-concentration corrosives may cause extensive damage to the eye if they hit the cornea with great force. The resulting superficial corneal damage leads to direct stromal contact with the corrosive agent [22, 23]. A combination of acid burns with ocular contusion has been described for exploding modern car batteries [24, 25]. Similarly, the osmolarity gradient plays a major role in the propagation and progression of tissue damage caused by chemical burns [3, 26–28].

Alkaline agents, in general, penetrate more deeply than acids. The hydroxyl ion causes saponification of fatty acids in cell membranes which results in cellular disruption [19]. Once the epithelium is compromised, alkaline solutions penetrate more rapidly into the underlying tissues, destroying proteoglycan ground substance and the collagen matrix. If the agent reaches the collagen fibrils of the trabecular meshwork, it can cause scarring inhibiting aqueous outflow, leading to secondary glaucoma. Strong alkaline agents penetrate into the anterior chamber and cause widespread inflammation of iris, lens, and ciliary body [7, 19]. Acids can denature proteins and cause coagulation necrosis, forming a barrier which can reduce further tissue penetration [6, 7, 29]. As mentioned earlier, hydrofluoric acid may exceptionally penetrate as readily as alkaline agents creating the same spectrum of injuries [3–5]. It should be emphasized that while acidic agents cannot penetrate as quickly and readily as alkaline agents, they are nonetheless quite capable of causing severe damage to the ocular surface.

The lysis of cell membranes liberates mediators of chemotaxis and inflammation such as prostaglandins, leukotrienes, and interleukins leading to an immediate immunological response [30, 31]. The uniform initial clinical picture does not follow a common chemical or physical mechanism but rather is the reflection of a general disturbance of corneal hydration, protein content, and cell vitality [3]. Subsequent progression of the injury and the healing process may fall anywhere between a highly active inflammatory process to a hyporeactive nonviable process due to complete tissue necrosis [3, 8, 30–32].

## 6. Clinical Course

The clinical course of ocular chemical injury can be divided into immediate, acute, early reparative (8–20 days), and late reparative phases [33].

The immediate phase begins from the moment a chemical agent comes in contact with the ocular surface [1, 33]. The key elements for determining the extent of chemical ocular injury and prognosis consist of the total area of the corneal epithelial defect, the area of the conjunctival epithelial defect, the amount of clock hours or degrees of limbal blanching, the area and density of corneal opacification, and increased IOP on presentation and loss of lens clarity [1, 15–17, 33].

The first seven days after chemical eye injury constitute the acute phase of recovery. During this time, the tissues clear themselves of contaminants while reestablishing the superficial protective layer of corneal epithelium. Significant inflammatory mechanisms begin to evolve on the ocular surface and the anterior chamber [1, 33]. In this stage, there is usually a rise in the IOP in a bimodal manner [8].

Early reparative phase, 8–20 days after the injury, is the transition period of ocular healing, in which the immediate regeneration of ocular surface epithelium and acute inflammatory events give way to chronic inflammatory response, stromal repair, and scarring [1, 33]. A persistent epithelial defect can lead to corneal ulceration during this stage. It has been attributed to action of digestive enzymes such as collagenase, metalloproteinase, and other proteases released from the polymorphonuclear leukocytes and the healing epithelium [34–40].

Three weeks after a chemical injury, the healing process continues with so-called late reparative phase. This stage is characterized by completion of healing with good visual prognosis and complications in those with guarded visual prognosis [1, 33]. A chronic, severe inflammatory reaction is often triggered by breakdown products of the damaged ocular tissue that act as new antigens, causing invasion of leukocytes and macrophages [30, 31]. In severe cases, this may involve the eyelids, the peripheral vitreous, and the retina [41]. Treatment-resistant secondary glaucoma is a frequent complication, requiring surgical intervention and long-time treatment with antiglaucomatous medications [3, 41]. Corneal scarring, xerophthalmia, ankyloblepharon uveitis, cataract, symblephara, cicatricial entropion or ectropion, and trichiasis may occur subsequently [42–46].

## 7. Management of Chemical Burns

Care of chemical burns essentially echoes both the basic mechanism of the initial incident and the subsequent inflammatory response.

### 7.1. Emergency Therapy

Immediacy of treatment influences the final outcome favorably; hence, one should not delay the treatment waiting for careful assessment of the injury. After an acute chemical burn, immediate and extensive irrigation is necessary to wash out the offending chemicals [6, 19, 26, 29, 47–49]. It is suggested to continue rinsing the eye for no less than 10 minutes [3]. Irrigating contact lenses including Morgan Lens can also be used to provide ocular irrigation and/or medication to the cornea and conjunctiva after chemical burn [50]. Commonly, the ocular surface pH is checked using a urinary pH strip and irrigation is continued until pH normalizes to 7. Universal systems like amphoteric solutions (mostly Diphoterine) have less exothermic reactivity in addition to nonspecific binding capacity to bases and acids which makes them appropriate solutions for emergency neutralization [51–53]. Any remaining particles are removed from the ocular surface with a moist cotton tip or fine-tipped forceps. Successful first line management of eye burns and adequate training of nonophthalmological emergency teams are imperative to ensure the best possible outcome. It is shown that prognosis is closely related to the efficiency of the immediate treatment measures [3, 54].

### 7.2. Acute Phase Treatment

The treatment plan largely depends on the examination findings. The main objectives of acute phase treatment are to foster reepithelialization, decrease inflammation, prevent infection, avoid further epithelial and stromal breakdown, and minimize the sequela.

### 7.3. Promoting Reepithelization

Preservative free tear substitutes and lubricating ointment can ameliorate persistent epitheliopathy, reduce the risk of recurrent erosions, and accelerate visual rehabilitation [1]. Generally, burn patients benefit from systemic ascorbic acid which may promote collagen synthesis and wound healing [36, 55, 56]. Autologous serum tears which contain many factors that promote healing may be used to promote epithelialization [57–63]. Likewise, bandage contact lenses may be considered for delayed epithelial healing. Large-diameter gas-permeable scleral contact lenses, such as the prosthetic replacement of ocular surface ecosystem (PROSE) (originally called the Boston Scleral Lens), have been used after chemical or thermal injury in an inpatient setting [7, 64–67]. They can also protect the cornea from desiccation and friction of the eyelids via blinking [68].

### 7.4. Anti-Inflammatory Therapy

Topical corticosteroids play a critical role in controlling acute inflammation after chemical injuries. They reduce inflammatory cell infiltration and stabilize neutrophilic cytoplasmic and lysosomal membranes. They also help resolving anterior chamber as well as conjunctival inflammation [69]. The downside is that they also inhibit reepithelialization and collagen synthesis. The conventional belief is that topical steroids should not be used beyond 10 to 14 days, as they increase the risk of inhibition of collagenesis, worsening corneal thinning, and possible corneal perforation in alkali burns [70, 71]. However, this is primarily a concern in severe injuries with persistent epithelial defects; otherwise, corticosteroids can (and should) be used safely beyond 7–10 days if the epithelium has already closed [15, 72].

Citrate has been used successfully to prevent polymorphonuclear leukocyte migration into the burnt tissue, thus reducing the release of free radicals and proteolytic enzymes [36, 55, 56]. Free radicals are formed by hydroxyl ions and may be scavenged by ascorbic acid and tocopherols [3]. Cycloplegic drops can be considered to blunt the pain from iris-ciliary body spasm [19].

### 7.5. Treatment of High Intraocular Pressure

As mentioned, alkali injuries that reach the trabecular meshwork can lead to elevated intraocular pressure which can be easily overlooked [73]. To minimize toxicity to the epithelium, oral aqueous suppression is generally preferred over topical agents.

### 7.6. Sequelae Prevention

The ocular surface should be inspected daily for symblepharon formation. A symblepharon ring can be placed in the fornices to effectively prevent symblepharon formation [7]. The largest size is preferable which provides good separation of the palpebral conjunctiva from the bulbar conjunctiva. Although the above measures can successfully prevent symblepharon formation in the acute phase, they cannot prevent the chronic cicatricial changes that lead to the formation of scarring and adhesions [74].

Corneal ulceration and melting tend to occur in the most severe injuries. Corneal thinning is potentiated by collagenases or matrix metelloproteinases, released from polymorphonuclear cells and other resident cells [75]. Proteinase inhibitors such as Aprotinin and collagenase inhibitors such as cysteine, acetylcysteine, sodium ethylenediamine tetra acetic acid (EDTA), calcium EDTA, penicillamine, citrate, and especially tetracyclines were found to prevent corneal thinning in chemically burned corneas [1, 19, 35, 75–77]. Systemic tetracycline may also boost healing of persistent corneal epithelial defects [7, 34].

## 8. Surgical Management

The primary intention of early surgery in the face of a chemical ocular burn is to maintain the globe and promote reepithelialization. Surgical management starts with initial debridement of the necrotic material and continues with amniotic membrane transplantation and tectonic grafting if necessary. Late surgical interventions, on the other hand, are aimed at restoring the normal ocular surface anatomy and visual function. These include correcting eyelid abnormalities, management of glaucoma, limbal stem cell transplantation, and ultimately keratoplasty.

### 8.1. Amniotic Membrane Transplantation

Amniotic membrane transplantation (AMT) can be used both as a graft which can provide a basement membrane for epithelialization and/or as a patch where it acts as a biological bandage contact lens [78–80]. It was shown that cryopreserved amniotic membrane transplantation to the entire ocular surface within two weeks of a chemical or thermal burn results in immediate pain relief and healing of epithelial defects in patients with grade II-III burns [81]. In addition, it is often used as an adjunct to medical therapy to decrease ocular surface inflammation and reduce scarring [2, 9, 79, 81–95]. Besides, multilayered AMT is an appropriate surrogate in severe corneal thinning [96, 97]. Further, amniotic membrane may also be applied to the cornea using a contact lens type carrier (ProKera, Bio-Tissue, Inc., Miami, FL) with the amniotic membrane being secured to a flexible plastic ring [11, 98]. The ring-amniotic membrane complex is placed onto the ocular surface, without any need for suturing or gluing. The amniotic membrane usually lasts days to weeks (typically around one week); however, its application can be repeated. Furthermore, AMT may be used as an adjunct to different techniques of stem cell transplantations if those procedures are indicated in the course of the treatment [11, 13, 14, 46, 82, 83, 98–103].

### 8.2. Tenonplasty

In severe, grade IV injuries, the loss of limbal vascularity may lead to anterior segment necrosis in addition to lack of reepithelialization and subsequent conjunctivalization of the cornea. Early intervention to reestablish the limbal blood supply may potentially prevent late complications [104]. Tenonplasty involves debridement of necrotic tissue and advancing viable, vascular Tenon's layer to the limbus securing it to sclera, combined with AMT with or without lamellar corneal patch grafting (Figure 1). It has been shown to prevent further scleral ischemia and melting [3, 104, 105].

### 8.3. Limbal Stem Cell Transplantation

Limbal stem cells deficiency is one of the most visually significant long-term sequelae of severe chemical injuries. Patients suffering from chronic irritation persistent epithelial defects with clinical signs of corneal conjunctivalization may be considered for stem cell transplantation [11, 12, 14, 103, 105–109]. In general, it is best to delay limbal stem cell transplantation (from the time of injury) as much as possible, since the more the ocular surface inflammation is controlled, the better the results would be. Likewise, it is advised to have all eyelid abnormalities (e.g., trichiasis and symblepharon) addressed before considering limbal stem cell transplantation [14, 45, 46, 103, 106, 110, 111].

**Figure 3:**
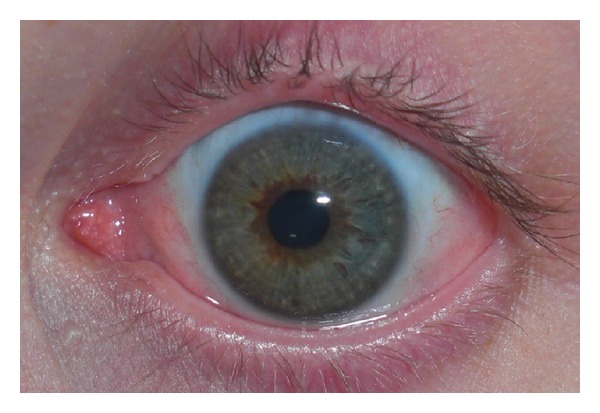
Patient with total limbal stem cell deficiency after chemical burn who was successfully treated with conjunctival-limbal autograft (2 years after surgery).

**Figure 4:**
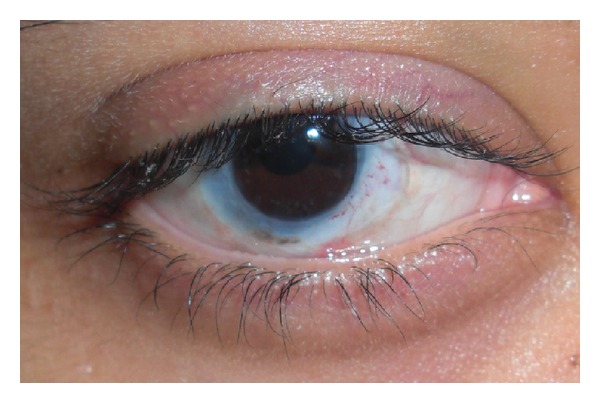
Patient with total limbal stem cell deficiency after chemical burn who underwent keratolimbal allograft and penetrating keratoplasty with systemic immunosuppression (18 months after surgery).

Limbal stem cells can be harvested from the patient (conjunctival-limbal autograft (CLAU) [44] and cultivated limbal epithelial transplantation (CLET) [43]), immediate family members including parents, siblings, or children (living-related conjunctival-limbal allograft (lr-CLAL)), or cadaveric eyes (keratolimbal allograft (KLAL)). Several surgical techniques have been described [14, 99, 112–115]. CLAU is only possible in unilateral burns but invariably has excellent results, with complete regression of corneal neovascularization such that successful reepithelialization and functional vision are achieved in 80–90% of patients (Figure 3) [44, 116]. CLET is a very suitable surgical alternative in cases with total unilateral LSCD [43]. In patients with bilateral ocular surface injury, lr-CLAL or KLAL are the available options. Harvesting tissue from one eye or both eyes of a first-degree relative provides fresh tissue with closer genetic composition. On the other hand, KLAL graft is more accessible with more stem cells because of larger clock hours of graft tissue available (Figure 4). Lr-CLAL also has the advantage of providing viable conjunctival tissue which may be used in patients with severe conjunctival deficiency. Systemic immunosuppression consisting of short-term steroids, tacrolimus (or cyclosporine), and mycophenolate (or azathioprine) is necessary to prevent limbal allograft rejection [14, 106, 110, 111]. Close collaboration with an organ transplant team is generally needed for the optimal management of the immunosuppression and monitoring of side effects [117].

### 8.4. Corneal Transplantation

Tectonic penetrating keratoplasty (PKP) which is a surgical intervention of last resort in burn patients may be inevitable in cases with severe thinning, large descemetoceles, and impending or frank corneal perforation. Conventional lamellar keratoplasty (LKP) or deep anterior lamellar keratoplasty (DALK; Melles and Anwar techniques) can be performed for visual rehabilitation of patients with extensive stromal scarring [118, 119]. Most often, due to corneal scar formation and variability of corneal thickness and irregularity, conventional LKP and Melles techniques are preferred [118]. Otherwise, full thickness transplants can be performed successfully, once the limbal stem cell deficiency has been addressed [120].

### 8.5. Keratoprosthesis

Artificial corneas undoubtedly can improve vision but should be considered in cases when PKP has failed or expected to fail (e.g., in the setting of extensive stromal vascularization) [121–126]. Currently, the Boston keratoprosthesis remains the main option in patients in which it has not been possible to restore corneal clarity and a normal ocular surface with any of previous measures [121, 127]. Their long-term risks, the need for life-long regular followups, and adherence to daily antibiotic prophylaxis are some of the issues that may make some patients less than ideal candidates for keratoprosthesis [122, 124, 125]. The Boston keratoprosthesis study group found excellent anatomical retention in patients with a chemical burn [122]. Reported long-term complications include retroprosthetic membrane formation, intraocular pressure elevation and/or glaucoma progression, sterile corneal stromal necrosis or corneal thinning, infectious keratitis, persistent epithelial defect, retinal detachment, sterile uveitis/vitritis, and infectious endophthalmitis [128–130]. The osteo-odonto-keratoprosthesis (OOKP) surgery is one of the last resorts usually kept for patients with bilateral corneal blindness resulting from several ocular and systemic pathologies [131]. Indications include severe end-stage Stevens-Johnson syndrome, Lyell's syndrome, epidermolysis bullosa, severe trachoma, chemical or physical injury, loss of lids, and multiple corneal graft failure. Other surgical alternatives available for treatment of such cases (e.g., ocular surface reconstruction with stem cell transplant) should be considered prior to OOKP surgery [132].

## 9. Conclusion

Chemical burns can have devastating consequences for the ocular surface and periocular structures. They frequently cause chronic pain, disfigurement, and vision loss. The overall goal of treatment is restoration of the normal ocular surface anatomy which starts with intensive treatment in the beginning and ultimately complex surgical procedures later in the course. With advancements in regenerative medicine, the clinical outcomes are expected to improve further.

## Figures and Tables

**Table 1:** Roper-Hall classification for the severity of ocular surface burns.

Grade	Clinical findings		Prognosis
	Cornea	Conjunctiva/limbus	
I	Corneal epithelial damage	No limbal ischemia	Good
II	Corneal haze, iris details visible	<1/3 limbal ischemia	Good
III	Total epithelial loss, stromal haze, and iris details obscured	1/3–1/2 limbal ischemia	Guarded
IV	Cornea opaque, iris and pupil obscured	>1/2 limbal ischemia	Poor
